# E-cadherin promoter hypermethylation may contribute to protein inactivation in pterygia

**Published:** 2010-06-09

**Authors:** Chi-Hsien Young, Yu-Te Chiu, Tung-Sheng Shih, Wan-Ru Lin, Chun-Chi Chiang, Ying-Erh Chou, Ya-Wen Cheng, Yi-Yu Tsai

**Affiliations:** 1 Institute of Medicine, Chung Shan Medical University, Taichung, Taiwan; 2 Institute of Occupational Safety & Health, Taipei, Taiwan; 3 Department of Ophthalmology, China Medical University Hospital, Taichung, Taiwan; 4 Graduate Institute of Clinical Medical Science, China Medical University and Hospital, Taichung, Taiwan; 5 Graduate Institute of Environmental Health, College of Public Health, China Medical University and Hospital, Taichung, Taiwan

## Abstract

**Purpose:**

Our recent reports indicated that the molecular changes of pterygia are similar to tumor cells. We believe that pterygia may have a similar mechanism in oncogenesis. Many studies have revealed that E-cadherin associated protein expression decreases in many tumors and pterygia. E-cadherin may be a marker for both tumor metastasis and prognosis. However, no studies have examined the reason for E-cadherin protein inactivation in pterygia. Therefore, this study aimed to analyze the association of E-cadherin promoter hypermethylation with protein inactivation in pterygial tissues.

**Methods:**

E-cadherin methylation-status and the expression of E-cadherin and β-catenin protein were studied using methylation-specific PCR and immunohistochemistry, respectively, on 120 pterygial specimens and 30 normal conjunctivas.

**Results:**

Hypermethylation of E-cadherin gene promoter was detected in 32 (26.7%) of the 120 pterygial specimens. A total of 79 (65.8%) pterygial specimens tested positive for E-cadherin protein expression and 41 (34.2%) specimens tested negative. The E-cadherin staining was limited to the membrane of the epithelial layer. There was a reverse correlation between E-cadherin gene promoter hypermethylation and E-cadherin protein expression (p<0.0001). Aberrant localization of β-catenin was higher in the E-cadherin negative group than in E-cadherin positive group.

**Conclusions:**

Our study demonstrates E-cadherin gene promoter hypermethylation were associated with low or absent expression of E-cadherin. Moreover, loss of E-cadherin protein may contribute to aberrant localization of β-catenin. These data provide evidence that methylation exists in pterygia and may play a role in their development.

## Introduction

Pterygium has long been considered a degenerative disease. However, following the finding that the p53 protein is abnormally expressed in epithelium, pterygium is now considered to be a UV exposure-related uncontrolled cell proliferation that is similar to a tumor [1-7].

There are several adhesion molecules, including cadherin, cell-cell adhesion molecules (CAMs), selectin, and integrin, which maintain tissue architecture [8]. E-cadherin (120 kDa; human chromosome 16q), a transmembrane protein, plays a role in organ morphogenesis, tissue formation, and proper development during embryogenesis [9]. The extracellular domain of E-cadherin connects neighboring cells with adherent junctions through calcium-dependent homophilic interactions [9]. The cytoplasmic part of E-cadherin links the cytoskeleton via α-catenin, β-catenin, and p120ctm [10]. β-catenin not only contributes to cellular adhesion but is also a central component in the Wingless/Wnt (Wg/Wnt) signaling cascade, which is important in axis determination and organogenesis during early development [11,12]. Without a mitotic signal, free cytoplasmic β-catenin would be degraded by the ubiquitin-proteasome system via phosphorylation by the protein complex, which is composed of adenomatous polyposis coli (APC) tumor suppresser protein, Axin, and serine threonine glycogen synthetase kinase (GSK-3β) [13]. This mechanism maintains a low level of free cytoplasmic β-catenin. However, free cytoplasmic β-catenin will increase with the appearance of mitotic signals (Wnt protein) or reduced expression of E-cadherin. The binding of the Wnt protein to its cognate frizzled receptor leads to activation of the dishevelled (Dsh) protein, which down-regulates the APC-Axin- GSK-3β protein complex [14]. Hence, cytoplasmic β-catenin evades degradation and accumulates in the cytoplasm [15,16]. In addition, reduced expression of E-cadherin will lead to the decomposition of the E-cadherin-catenin complex and to an increase in free cytoplasmic β-catenin. E-cadherin also mediates epithelial cellular adhesion. When the free cytoplasmic β-catenin increases, it eventually translocates into the nucleus and binds with transcription factors LEF (Lymphoid Enhancer Factor) and TCF (T Cell Factor) resulting in the activation of target genes [17,18]. The target genes, such as cyclin D1 and c-myc, are responsible for cell proliferation and neoplastic transformation [19,20]. Therefore, E-cadherin contributes to epithelial differentiation.

Methylation of gene regulatory elements, a well known epigenetic change, acts as an important alternative to genetic alteration for gene inactivation. Aberrant methylation of the promoter region of tumor suppressor genes and the associated gene silencing plays an important role in the pathogenesis of most, if not all types of human cancer [21]. The methylation of CpG islands in the promoter region silences gene expression and is a normal event that occurs in cells to regulate gene expression. However, the mechanisms involved in hypermethylated DNA loci remain unclear. CpG island hypermethylation of the E-cadherin gene (CDH1) promoter is a contributing factor in the inactivation of E-cadherin [22]. E-cadherin promoter hypermethylation is involved in many types of cancers [9].

In this study, we hypothesized that the E-cadherin gene loses function in pterygium. We also hypothesized that besides gene mutations, there are epigenetic changes (e.g., hypermethylation of gene regulatory elements) in pterygium. To test these hypotheses, we analyzed hypermethylation of the E-cadherin gene promoter in pterygium and the relationship between hypermethylation and E-cadherin protein expression. In addition, the association of E-cadherin protein with localization of β-catenin was analyzed in this study.

## Methods

### Patients and controls

Pterygial samples were harvested from 120 patients undergoing pterygium surgery and the patients were asked to submit a written informed consent approved by the Institutional Review Board. Patients in whom the apex of the pterygium had invaded the cornea by more than 1 mm were included in this study. The controls included normal conjunctival samples collected from the superior conjunctiva of 15 patients and the medial conjunctiva of 15 patients without pterygium and pinguecula who were undergoing cataract or vitreoretinal surgery. There were 70 males and 50 females in the pterygium group (age range=52–78 years, means=62.5 years), and there were 15 males and 15 females in the control group (age range=55–75 years, mean=62.8 years). Normal conjunctival samples were collected from bulbar conjunctivas. All pterygial specimens came from primary pterygium.All specimens were fixed in formalin and paraffin embedded.

### Immunohistochemistry

All sections were deparaffinized in xylene, sequentially rehydrated in alcohol and washed in phosphate-buffered saline. Sections used for E-cadherin and β-catenin, detection were heated in a microwave oven twice for 5 min in a citrate buffer (pH 6.0). Mouse anti- E-cadherin and a β-catenin monoclonal antibody (at a dilution of 1:200) (Santa Cruz Biotechnology, Inc., Santa Cruz, CA) were used as the primary antibodies. The sections were incubated with the primary antibodies for 60 min at room temperature, and the signals were detected by using a conventional streptavidin peroxidase method (LSAB kit K675; Dako, Copenhagen, Denmark). Signals 

were developed with 3,3'-diaminobenzidine for 5 min and counterstained with hematoxylin. The detailed protocol used has been described in our previous reports [23]. Negative controls that did not include the primary antibodies were set up. Conjunctiva tissues and lung epithelial cells were used as the positive control. The results were evaluated independently by three observers and were scored for the percentage of positive expression. In E-cadherin protein, which was only expressed in the membrane, we counted the positive rate of membrane expression of total epithelial cells in β-catenin, which were expressed in the membrane, cytoplasm, and nuclei. We separated the expression site to two groups, membrane expression and cytoplasm/nuclei expression. Score 0, no positive staining; score +, from 1% to 10%; score ++, from 11% to 50%; and score +++, more than 50% positive cells. In this study, scores of ++, and +++ were considered to be positive immunostaining, and a score of 0 or + was classified as negative immunostaining.

### Methylation-specific PCR (MSP) and direct sequencing

For bisulfite treatment, 2 μg of DNA was diluted in 50 μl water and denatured in 0.2 M NaOH at 37 °C for 20 min. Sodium bisulfite NaHSO_3_ (130 μl; 3.5 mol/l; freshly prepared; Sigma-Aldrich, Inc., St. Louis, MO) and hydroquinone (10 μl; 30 mmol/l; freshly prepared; Sigma) were added to the samples and incubated at 95 °C for 5 min and 50 °C for 16 h. After bisulfate modified, DNA were purified using the Wizard DNA Clean-Up System (Promega Corp., Madison, WI) according to the manufacturer’s instructions. The purified DNA was mixed with 0.6 N NaOH to a final concentration of 0.3 N, incubated for 10 min at room temperature, and then ethanol precipitated. The DNA was then re-suspended in 10–15 μl ddH_2_O and stored at −20 °C until it was used for PCR. PCR was performed using primers specific for the methylated E-cadherin sequence. Sense and antisense primers for the methylated sequence were 5′-TGT AGT TAC GTA TTT ATT TTT AGT GGC GTC-3′and 5′-CGA ATA CGA TCG AAT CGA ACC G-3′, respectively. All bisulfate-treated DNA was also amplified using primers specific to the unmethylated E-cadherin sequence. Sense and antisense primers for the unmethylated sequence were 5′-TGG TTG TAG TTA TGT ATT TGT TTT TAG TGG TGT T-3′ and 5′-ACA CCA AAT ACA ATC AAA TCA AAC CAA A-3, respectively. The PCR consisted of an initial denaturation step at 95 °C for 5 min followed by 40 cycles at 94 °C for 60 s; 65 °C (for methylated E-cadherin)/60 °C (for unmethylated E-cadherin) for 50 s; 72 °C for 50 s; and a final extension step at 72 °C for 10 min. PCR products were analyzed by agarose gel electrophoresis and visualized using ethidium bromide staining. Bisulfite-modified DNA from peripheral blood lymphocytes of a healthy individual served as a positive control for the unmethylated alleles, and unconverted DNA from normal lymphocytes was used as a positive control for the methylated alleles.

### Statistical Analysis

Statistical analysis was performed using the SPSS statistical software program (SPSS Inc., Chicago, IL). The Fisher’s exact test was applied for statistical analysis. A p<0.05 was considered to be statistically significant.

## Results

### E-cadherin protein expression in pterygium

**Figure 1:**
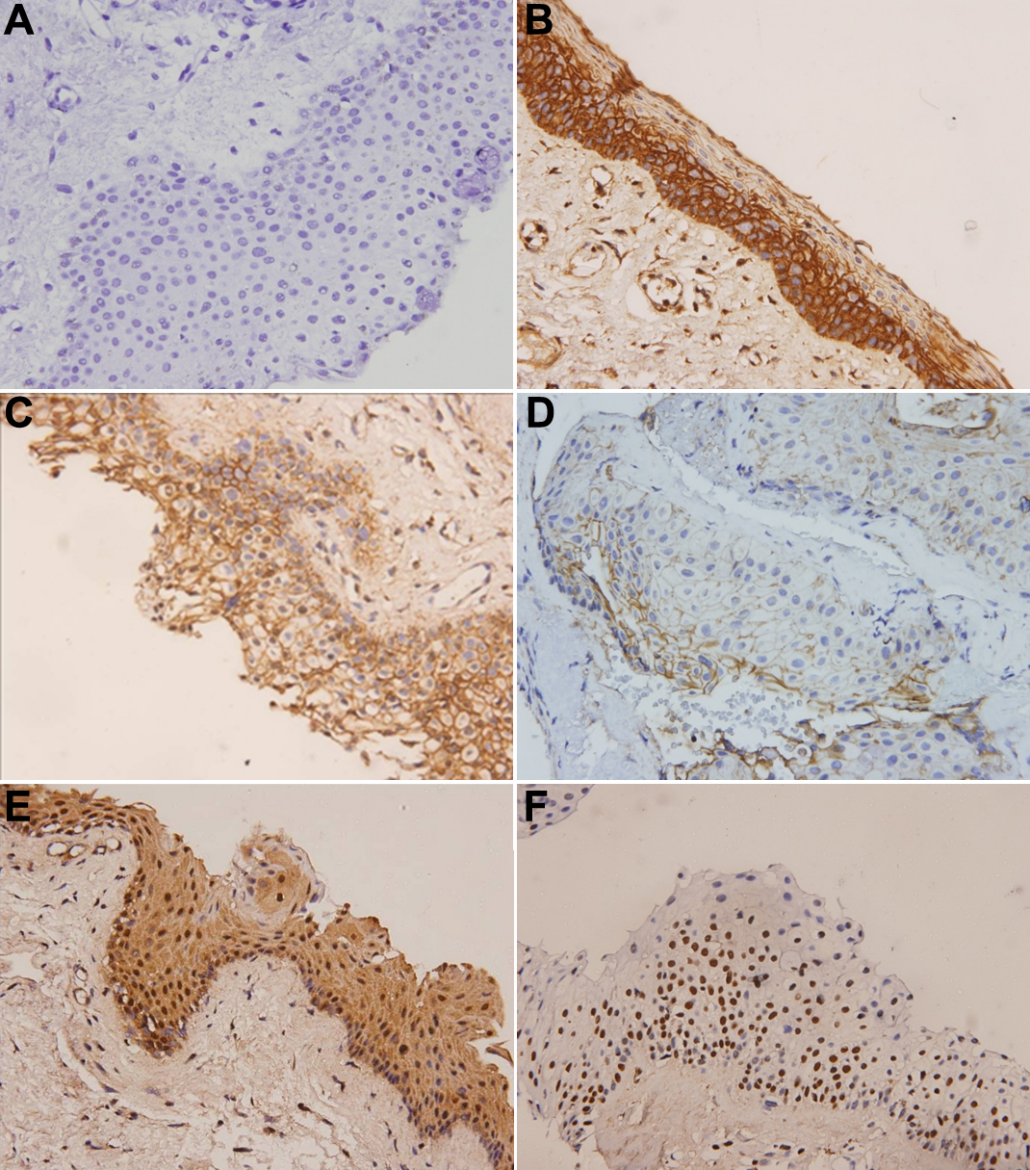
Representative immunostaining results for E-cadherin associated protein expression. **A**: The first antibody replaced with IgG was used as the negative control. **B**: The representative positive β-catenin immunostaining in conjunctival tissue was used as the positive control. **C**: E-cadherin protein expression detected in the membrane (400×). **D**: β-Catenin protein expression detected in the membrane (400×). In addition, aberrant localization of β-catenin was also detected in the cytoplasm/nuclei (**E**) and nucleus (**F**) (400×).

Immunostaining scores of 0 and + were considered negative, and ++ and +++ were considered positive (cutoff level set at 10%). In the pterygium group, the E-cadherin, proteins were detected in 79 (65.8%) patients (Table 1). E-cadherin was positive for immunostaining in the membrane (Figure 1).

**Table 1:** E-cadherin and β-catenin protein expression in pterygium analyzed by immunohistochemistry.

** **	**Pterygium**		**Control**	
**Protein**	**N**	**%**	**N**	**%**
**E-cadherin**				
Negative	41	34.2	0	0
Positive	79	65.8	30	100
**β-catenin**				
Negative	62	51.7	0	0
Positive	** **	** **	** **	** **
Membrane	33	27.5	30	100
Nuclei/Cytoplasm	25	20.8	0	0

### Loss of E-cadherin protein expression in pterygium may correlate with promoter methylation

To understand the reason for negative expression of E-cadherin in pterygium, MS-PCR was used to analyze E-cadherin promoter hypermethylation. The relationship between E-cadherin gene promoter hypermethylation and E-cadherin protein expression is shown in Table 2. Of the 32 pterygium cases with E-cadherin gene promoter hypermethylation, 21 (65.6%) were negative for E-cadherin protein expression, which was higher than the pterygium cases without E-cadherin gene promoter hypermethylation (22.7%). There was a significant reverse correlation between E-cadherin gene promoter hypermethylation and E-cadherin protein expression (p<0.0001; Table 2).

**Table 2:** Relationship between E-cadherin gene promoter hypermethylation and protein expression in pterygium.

** **	** **	**Protein**		** **
**E-cadherin**	**Total**	**Negative N (%)**	**Positive N (%)**	**p value**
Promoter methylation				
Negative	88	20 (22.7)	68 (77.3)	** **
Positive	32	21 (65.6)	11 (34.4)	<0.0001

### β-catenin protein expression in pterygium

As we know, reduced expression of E-cadherin will lead to the decomposition of the E-cadherin-catenin complex and to an increase in free cytoplasmic β-catenin. When the free cytoplasmic β-catenin increases, it finally translocates into the nucleus resulting in the activation of cyclin D1 and c-myc which are responsible for cell proliferation and neoplastic transformation [19,20]. We further analyzed the correlation of E-cadherin and β-catenin protein expression in pterygium. In the pterygium group, the β-catenin proteins were detected in 58 (48.3%) patients (Table 1). Additionally, as shown in Figure 1, the aberrant protein localization of β-catenin was also detected in pterygium. In 58 β-catenin positive patients, 56.9% (33 of 58) were detected in the membrane and 43.1% (25 of 58) were detected in cytoplasm or nuclei. In the normal conjunctiva group, all specimens were positive for immunostaining in the membrane.

### Correlation of E-cadherin and β-catenin protein expression

As we know, the extracellular domain of E-cadherin connects neighboring cells with adherent junctions through calcium-dependent homophilic interactions and the cytoplasmic part of E-cadherin links the cytoskeleton via β-catenin [9]. To understand the relationship between the E-cadherin signaling pathway and associated protein expression, the correlations of E-cadherin and β-catenin protein expression and localization were analyzed (Table 3). As shown in Table 3, in the 41 cases of E-cadherin negative expression, 43.9% (18 of 41) had β-catenin negative expression, 7.4% (3 of 41) had β-catenin membrane expression, and 48.7% (20 of 41) had nuclei/cytoplasm expression. Additionally, in the 79 cases of E-cadherin positive expression, 55.7% (44 of 79) had β-catenin negative expression, 38.0% (30 of 79) had β-catenin membrane expression, and 6.3% (5 of 79) had nuclei/cytoplasm expression. The aberrant protein localization of β-catenin in the E-cadherin negative group was significantly higher than in the positive group (p<0.0001).

**Table 3:** Relationship of E-cadherin protein expression and β-catenin protein localization in pterygium.

** **	**E-cadherin**		** **
**β-catenin**	**Negative (n=41; %)**	**Positive (n=79; %)**	**p value**
Negative (n=62)	18 (43.9)	44 (55.7)	** **
Positive			
Membrane (n=33)	3 (7.4)	30 (38.0)	** **
Nuclei/Cytoplasm	20 (48.7)	5 (6.3)	<0.0001

## Discussion

Pterygia, previously considered a degenerative process of the corneal limbus, is characterized by the invasion of a fleshy triangle of conjunctival tissue onto the cornea. Shimmura et al. [24] demonstrated that increased activity of telomerase in pterygia epithelial cells, indicating their hyperproliferative nature. Satoru et al. [25] reported that increased expression of Ki67, a cell proliferation marker, was noted in pterygia. We assume that it is the proliferative capacities of pterygial cells which make pterygia appear to have a similar mechanism to tumorigenesis.

Cadherins have been postulated to be responsible for the occurrence of contact inhibition of cell growth [9]. Altered expression of E-cadherin may result in the loss of contact inhibition and abnormal cell proliferation, hence tumorigenesis takes place [26,27]. In this study, reduced expression of the E-cadherin expression was observed by immunohistochemistry (IHC) staining. Previous reports indicated that the nuclear β-catenin associated with TCF/LEF proteins will activate the target genes, such as cyclin D1 and c-myc [19,20,28], leading to cellular proliferation and division [19,20,28,29]. The nuclear β-catenin involved in the progression of proliferation has been found in several types of cancers [30]. In pterygia, a previous report has indicated that the E-cadherin, as well as β-catenin, was heterogeneously expressed in the cell membrane and cytoplasm of pterygia. In addition, β-catenin immunoreactivity were also showed intense nuclear in several epithelial cells [31]. The results were similar with our finding. As shown in Figure 1, we not only found reduced protein but also aberrant protein localization of the β-catenin protein in pterygium. Additionally, the frequency of aberrant localization of β-catenin in the E-cadherin negative groups was significantly higher than in the positive groups. Therefore, we recognize that the changes in E-cadherin signaling pathway protein expression and localization are involved in pterygium cell proliferation.

Aberrant promoter hypermethylation of the tumor suppressor genes inactivates the gene function, and the resultant gene silencing plays an important role in the pathogenesis of most, if not all, types of human cancer [32]. In addition, the hypermethylation may act to freeze inactivated genes in the “off” position. However, the mechanisms involved in hypermethylating DNA loci remain unclear. The global cytosine methylation patterns in mammals appear to be established by a complex interplay of at least three independently encoded DNA methyltransferases (DNMTs), including DNMT1, DNMT3a and DNMT3b [33,34]. Our previous study showed that 29.5% of pterygial specimens tested positive for DNMT3b protein expression [35]. In the present study, MS-PCR was performed to analyze promoter hypermethylation of E-cadherin (CDH1). Fifty-one per cent of the pterygia without E-cadherin protein expression were associated with promoter hypermethylation. In addition, 13.9% of the pterygia with E-cadherin protein expression were associated with CDH1 promoter hypermethylation (p<0.0001, Table 2). Therefore, decreased expression of E-cadherin protein might be related to promoter hypermethylation.

A previous report indicated that Snail and Slug, repressors of E-cadherin transcription, inhibit the expression of E-cadherin [36]. The downstream cascade, including cytoplasmic accumulation and nuclear translocation of β-catenin, therefore results in epithelial mesenchymal transition (EMT) and cell proliferation of pterygium epithelial cells. Although the signaling pathways involving EMT after ligand activation are complex, the downregulation of E-cadherin plays a crucial role in pterygial pathogenesis [37]. Previous reports showed that Histone deacetylation is associated with transcriptional silencing of E-cadherin in colorectal cancer and ovarian cell lines [38,39]. Hence, factors, such as gene mutations, genomic deletions, Histone deacetylation, or polymorphisms causing inactivation of E-cadherin should be further investigated.

In conclusion, our study is the first to provide evidence to show that the promoter hypermethylation of E-cadherin may be involved in the reduction of E-cadherin protein expression in pterygium. Additionally, we also provided evidence for the detection of aberrant protein localization of β-catenin in pterygium. These data provide evidence that changes in E-cadherin signaling may contribute to pterygium cell proliferation.
